# GPX3-Mediated Oxidative Stress Affects Pyrimidine Metabolism Levels in Stomach Adenocarcinoma via the AMPK/mTOR Pathway

**DOI:** 10.1155/2024/6875417

**Published:** 2024-01-30

**Authors:** Yaowen Zhang, Yixin Yang, Shanshan Kuang, Yang Zhang, Hancheng Qin, Jisheng Xie

**Affiliations:** ^1^Department of Histology and Embryology, Youjiang Medical University for Nationalities, Baise, China; ^2^Department of Pathophysiology, Youjiang Medical University for Nationalities, Baise, China

## Abstract

**Background:**

Amino acid metabolism, including ATP production, nucleotide synthesis, and redox homeostatic processes, are associated with proliferation and differentiation of tumor cells. This study aimed to identify novel prognostic biomarkers and potential therapeutic targets of amino acid metabolism-related genes for stomach adenocarcinoma (STAD).

**Methods:**

RNA sequencing transcriptome data in the TCGA-STAD (training set) and GTEx datasets (validation set) were used. The LIMMA R program enabled the differentially expressed amino acid metabolism-related genes (AAMRGs) to be found. A prognostic risk score model based on clinical phenotypic features was built using LASSO regression and step multi-Cox analyses. Gene set enrichment analysis (GSEA) was used to find potential molecular pathways associated with STAD. Hierarchical cluster analysis was used to evaluate pyrimidine metabolism. Cultured STAD cells assessed the proliferation of STAD and upregulation of GPX3 expression by CCK8 and flow cytometry. Transwell and wound healing assays assessed the impact of GPX3 on invasion and migration of STAD cells. Western blot and qRT-PCR were used to measure changes in pyrimidine metabolism-related markers and active molecules involved in the AMPK/mTOR signaling pathway.

**Results:**

Three AAMRGs, DNMT1, F2R, and GPX3, could independently predict the course of STAD. Pyrimidine metabolism appeared to be significantly associated with these by GSEA and clustering analyses. Pyrimidine metabolism was negatively correlated with GPX3. Functional studies using an overexpressed GPX3 plasmid showed an enhanced migration and invasion of STAD cells as well as the expression of genes associated with pyrimidine metabolism and the AMPK/mTOR signaling pathway. By using a CAD siRNA, it was found that that GPX3 affected 5-fluorouracil resistance during *de novo* synthesis of pyrimidine through the CAD-UMPS signaling axis.

**Conclusions:**

GPX3 which regulates the level of pyrimidine metabolism through the AMPK/mTOR pathway was found to be closely associated with STAD. Our findings demonstrate GPX3 is a reliable biomarker for the prognosis of amino acid metabolism and a probable target for STAD therapy.

## 1. Introduction

Stomach adenocarcinoma (STAD) is the most prevalent type of digestive tumor. According to data from the International Agency for Research on Cancer (IARC), East Asia has the highest incidence and mortality rate of STAD in the world and almost half of the patients in China [1]. In China, STAD ranks third among cancer-related deaths, which greatly jeopardizes the health of Chinese residents [2]. STAD is a multifactorial and multiprocess malignant tumor in which many factors act on related genes and regulatory factors at different stages of the development of the disease [3, 4], causing abnormalities in the structure and expression levels of related genes [5–7]. Although STAD-related marker studies are constantly being explored, the processes involved remain incompletely understood, and thorough investigations still lack systematic analyses regarding genetic factors associated with its prognosis. Therefore, it is urgent and critical to find new molecular targets to control the growth and invasion of STAD cells.

Amino acids, the basic units of proteins, are essential for the biosynthesis of nucleotides, glutathione, glucosamine, and polyamines [8], and they drive the formation of tumors. Most oncogenic drivers are known to upregulate nucleotide biosynthesis capacity, and many of the aggressive properties of cancer cells [9], including uncontrolled proliferation, chemoresistance, immune escape, and metastasis, are largely dependent on enhancing their metabolism [10]. Nucleotide metabolic pathways include both purine and pyrimidine metabolism [11]. In recent years, it has been shown that purines act as signaling ligands in many human cancers and that pyrimidines are key factors in tumor cell proliferation [12]. Excessive synthesis and use of nucleotide triphosphates (NTPs) and their deoxy counterparts (dNTPs) in cancer cells allow for abnormal metabolic changes. In overactive nucleotide reactions, dNTPs also become rate-limiting factors in several fundamental biological processes, including DNA replication, repair, and transcription, and they are critical for cancer initiation and progression downstream of oncogene activation [13]. Although there are some drug sensitivity studies on NTPs and dNTPs [14–16], relatively few were with respect to targeting amino acid metabolism genes to regulate nucleotide metabolism. Therefore, this study was undertaken to investigate pyrimidine metabolism with a view to provide fresh perspectives on the diagnosis and therapy of STAD and the development of related drugs.

Glutathione peroxidase 3 (GPX3) is found in the 5q32 region of chromosome 5 and comprises of five exons that span 10 kb and encode a 23 kDA protein that forms a homotetramer [17]. GPX3, as the only exocrine member of the GPX family, has a significant impact on oxygen radical detoxification. Its serum level can also be an important marker for detecting tumors [18]. Reactive oxygen species (ROS) are highly reactive molecules that regulate important signaling pathways in the body [19]. A moderate increase in ROS leads to various pathological conditions in the body, including tumor development. ROS are also involved in different signaling pathways and induce DNA mutations [20]. In recent years, it has been shown that GPX3 may play a role in ROS generation, including the enhancement of lipid hydroperoxide production near lipoxygenase (LOX) on the cell surface [21, 22]. Hence, GPX3 may be involved in cancer by regulating the levels of ROS and may affect tumor progression by acting as a potent inhibitor of cancer development and progression. Although several experimental studies in recent years have shown that GPX3 can be used as a prognostic biomarker affecting the migration and invasion of STAD cells [23], its specific role in STAD remains to be investigated. There have also been reports that the AMPK/mTOR signaling pathway is crucial for controlling anticancer drug resistance [24] and basic cellular metabolism [25–27].

In this study, GPX3 was identified as a key target for regulating ROS in tumor cells, whose expression is downregulated in STAD and this could affect the prognosis of patients with this disease. Our data suggest that GPX3 mediates the AMPK/mTOR signaling pathway by regulating the level of ROS in tumor cells and triggering an imbalance of oxidative stress in the organism. This subsequently impacts pyrimidine metabolism levels and resistance to 5-fluorouracil (5-Fu), a drug used in chemotherapy. Our data would suggest that GPX3 can be a promising prognostic indicator as well as a potential therapeutic target for STAD.

## 2. Materials and Methods

### 2.1. Data Source

The data were obtained from the STAD dataset in The Cancer Genome Atlas (TCGA, https://www.cancer.gov/tcga) database, which contains 375 and 32 tissue and paraneoplastic tissue samples obtained from STAD patients. In addition, the whole gene transcriptome sequencing data and patient clinical information data are also available. The GSE84437 dataset from the Gene Expression Omnibus (GEO) database (https://www.ncbi.nlm.nih.gov/geo/) was then used to verify the analytic findings obtained from the TCGA database. The gene expression profiles were filtered and normalized using the Perl, EdgeR, and gplot R software packages. The “sva” package in R software was specifically designed to eliminate batch effects. Screening of differentially expressed genes (DEGs) between paracancerous and STAD tissues was performed with adjusted *P* values <0.01 and |log2(FC)| > 1). The expression of GPX3 was extracted using the Perl and LIMMA software packages. Amino acid metabolism-related genes (AAMRGs) were downloaded through the GSEA database (https://www.gsea-msigdb.org/gsea/login.jsp).

### 2.2. Independent Prognostic Analysis

Genes having a *P* value of less than 0.05 were screened for stomach cancer using UniCox regression analysis. From the 480 amino acid metabolism-related genes obtained, 59 were screened, and a gene expression matrix was constructed based on these for subsequent screening and construction of prognostic risk models.

### 2.3. Construction and Validation of Prognostic Risk Models

The final prognostic model was constructed by building stable AAMRGs after UniCox regression analysis had screened for those that were differentially expressed in STAD. The LASSO algorithm was then used to prevent the model from being overfitting, and a stepwise multi-Cox regression analysis was carried out to determine which prognostic genes were best for this model. The following formula, which combines the regression coefficients and the expression values for each AAMRG, was used to determine the risk score: (index gene expression 1 × gene1) + (index gene expression 2 × gene2) +…+ (index gene expression 10 × gene10). All treated STAD patients in the TCGA database were divided into two subgroups of high and low based on their median risk ratings. Kaplan–Meier curves were used to ascertain the prognostic variations among the groups. Then, using receiver operating characteristic (ROC) curves, the one-, three-, and five-year survival rates of the patients were determined. Finally, the prognostic risk model that was created was verified using the GEO dataset.

### 2.4. Evaluation of Prognostic Risk Models

The model was analyzed for differences by using R software to determine whether the AAMRGs involved in the model construction differed in the high- and low-risk groups of patients with STAD by plotting box line plots. To determine the difference in survival between the high- and low-risk groups, survival analysis was carried out using the Kaplan–Meier analysis with *P* < 0.05 being judged as statistically significant. Risk analysis was performed by using the “pheatmap” program in R software to see how the risk of the patients increased and how it was related to their gene and survival status as well as their risk score. The area under the ROC curve served as a proxy for the model's accuracy. The independence of the models was assessed by using univariate and multivariate COX regression analyses.

### 2.5. Construction and Validation of a Predictive Model for Column-Line Graphs

We combined the patients' clinical information, including age, gender, pathologic grade, clinical stage, and TNM stage, with risk scores to create a line graph prediction model using the “rms” package in R software to predict the survival of patients at different stages of the disease. We also generated calibration curves to show the agreement between the column-line graphs in predicting the patient survival and the actual patient survival at years 1, 3, and 5.

### 2.6. Gene Set Enrichment Analysis (GSEA)

Upon dividing the gene set data of the 407 patients in the TCGA database into risk-scored high- and low-risk groups, the enrichment pathways in the two subgroups were ascertained by using the GSEA software to analyze the “c2.cp.kegg.v7.1.symbols.gmt” collection that was obtained from the molecular signature database. The phenotype labels for high-risk and low-risk groups were applied, 1000 permutations were allowed and all other choices were left at their default settings. The top five biological processes in each subgroup which were substantially enriched were visualized using the R software's “ggplot2” tool. Data from the Kyoto Encyclopedia of Genomes (KEGG) database were selected to analyze the signaling pathways mediated by GPX3 in STAD using the single-gene GSEA enrichment analysis function in the Sangerbox 3.0 tool (https://sangerbox.com/login.html).

### 2.7. Non-Negative Matrix Factorization (NMF) Clustering

The KEGG pyrimidine metabolism gene set was used for NMF clustering. The “NMF” package in R software was used for unsupervised cluster analysis of AAMRG expression to create molecular subtypes of pyrimidine metabolism for genes associated with amino acid metabolism and to assess the prognosis of various subgroups. The optimum *K* values were determined using consensus heat maps and NMF rating surveys, and TCGA samples were divided into two groups according to the significance of pyrimidine metabolism. Kaplan–Meier survival analysis was used to investigate variations in the survival of patients between the clusters.

### 2.8. Single Sample GSEA (ssGSEA)

ssGSEA analysis was performed using the “GSVA” and “GSEABase” program in the R software package based on the KEGG pyrimidine metabolism genes in the GSEA database. The results were plotted by using the ggplot2 program of the R software.

### 2.9. Cell Culture and Transfection

The Chinese Academy of Sciences (Shanghai, China) provided the HGC-27 and SGC-7901 cells, and Guangzhou Saiku provided the GES-1 cells. The cells were revived and cultured with RPMI 1640 complete medium, which included 10% newborn fetal bovine serum and 1% penicillin-streptomycin. The cells were then incubated at 37°C in an atmosphere of 5% CO_2_ and the culture media replaced every two to three days. Plasmids (Genepharma, Suzhou, China) were transfected by using HighGene plus Transfection reagent (Abclonal, USA) to overexpress GPX3 in SGC-7901 cells. The transfection efficiency was confirmed by western blotting and real-time fluorescence quantitative polymerase chain reaction (qRT-PCR).

### 2.10. Cell Counting Kit-8 Assay

1.5 × 10^4^ cells/well were used to inoculate the 96-well plates, and the cells were kept at 37°C in an atmosphere of 5% CO_2_ for 24 hours. Ten microliters of CCK8 solution were added to each well at a designated time. After three hours, a multiscan spectrophotometer was used to measure absorbance at 450 nm.

### 2.11. Migration and Invasion Assays

The capacity of STAD cells to invade and migrate was evaluated by using a transwell membrane (Corning 3422, pore size 8 *μ*m) in the presence and absence of Matrigel. In summary, 200 *μ*L of FBS without medium was placed in the upper chamber of the transwell, and 2–4 × 10^4^ cells were put into it. Concurrently, the bottom chamber was filled with 500 *μ*L of culture media containing 10% FBS. After incubation for 24 hours at 37°C, the cells were fixed with 4% paraformaldehyde for 30 minutes and then washed with PBS. After scraping the cells from the top side of the membrane with a cotton swab, the cells were stained with crystal violet for 30 minutes at room temperature. After drying, the membranes were washed with PBS and imaged by using the ImageJ software.

### 2.12. Wound Healing Assay

A sterile plastic pipette was used to scrape the cell layer after cells were aliquoted into 6-well plates for the wound healing experiment. After that, cells were grown in media without FBS, and at 0 h and 48 h, respectively, electron microscopic images were captured. The cells' migratory potential was evaluated by calculating the changes in the size of the injured area.

### 2.13. Total RNA Isolation and Quantitative RT-PCR

The kit Axy Prep Multisource Total RNA Miniprep Kit (Suzhou Youyi Landi Biotechnology Co., Ltd.) was used to extract total RNA from cells. The cDNA was synthesized by reverse transcription according to the instruction steps of the reverse transcription kit MonScript RTIII All-in-One Mix with dsDNase (Mona Biotechnology Co., Ltd.). Reverse transcription was performed to synthesize cDNA, cDNA was used as a template and qRT-PCR was performed according to the instructions of the MonAmp SYBR Green qPCR Mix (None ROX) kit. GAPDH was used as an internal reference to calculate the relative expression of individual genes by using the 2^−ΔΔCt^ method. Each measurement was repeated three times.

### 2.14. Western Blotting Analysis

A protease inhibitor mixture-containing RIPA lysis buffer (Biyuntian Biotechnology, China) was used to lyse STAD cells. The protein content was determined using the BCA protein quantification kit (Vazame, China). Equivalent amounts of protein samples were separated using SDS-PAGE and then transferred onto PVDF membranes. Following 0.5 hours of equilibration in a protein-free quick closure solution (Vazame, China), the membranes were incubated with the relevant primary antibodies overnight at 4°C. Primary antibodies were purchased from ABclonal (GPX3, 1 : 2000; mTOR, 1 : 10000; P-mTOR, 1 : 10000), Proteintech Group (CAD, 1 : 5000; AMPK, 1 : 2000; P-AMPK, 1 : 1000), and ImmunoWay (GAPDH, 1 : 1000). After washing with PBS, the membranes were incubated for 1 hour at room temperature with HRP-labeled secondary antibodies. The secondary antibodies were purchased from Elabscience (goat anti-rabbit IgG (*H* + *L*), 1 : 1000; goat anti-mouse IgG (*H* + *L*), 1 : 1000). Following further washes with PBS and incubation with a chemiluminescence reagent, the membranes were visualized and analyzed by using the ImageJ software.

### 2.15. Detection of Reactive Oxygen Species in Cells

SGC-7901 cells were loaded using the DCFH-DA fluorescent probe from the Reactive Oxygen Species Assay Kit. The DCFH-DA probe stock solution was first diluted with serum-free culture medium at a ratio of 1 : 1000 to a final concentration of 10 mM, and then 2 mL of DCFH-DA dilution was added to each well of a six-well plate. The SGC-7901 cells were incubated for 20 min in a cell culture incubator at 37 degrees Celsius, and then the cells were washed three times with serum-free cell culture medium. Finally, laser confocal microscopy and a fluorescent enzyme marker were used to measure the amount of ROS in different groups of cells.

### 2.16. Statistical Analysis

We generated statistical visualizations of the data using GraphPad Prism8 and the ggplot2 R software package. In all cases, a *P* value of less than 0.05 was considered to be statistically significant.

## 3. Results

### 3.1. Identification and Screening of AAMRGs

**Figure 1:**
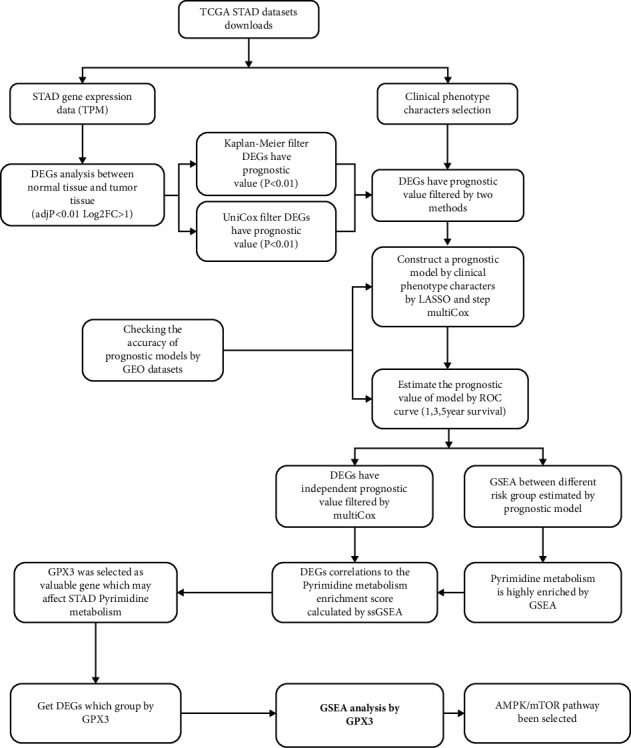
Flowchart of the research.

**Figure 2:**
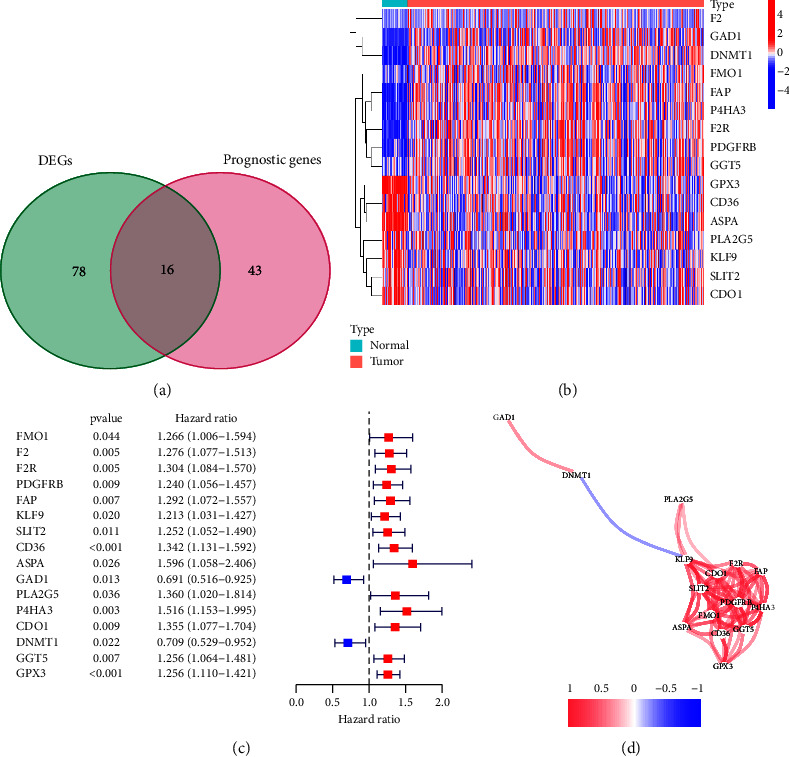
Screening of 16 AAMRGs. (a) The Venn diagram shows that 16 AAMRGs were selected after differential expression analysis and univariate COX regression analysis. (b) Heat maps showed the differential expression of these 16 AAMRGs in normal tissues and tumor tissues. (c) Through univariate COX regression analysis, these 16 AAMRGs can affect the prognosis of patients with gastric cancer. (d) The correlation between the 16 AAMRGs is shown through the network diagram.

Figure 1 diagrammatically illustrates the protocol used in the study. Using data from the TCGA-STAD cohort of the TCGA database, we first identified 94 AAMRGs expressed differently in gastric cancer tissues when compared to normal tissues. Next, we used the “survival” R package to perform univariate COX regression analysis to examine the 59 genes linked to the prognosis of STAD. 16 AAMRGs which were observed to be DEGs in STAD and normal gastric tissues, and these were considered to be capable of influencing the prognosis of STAD (with 9 and 7 AAMRGs highly and lowly expressed in STAD, respectively; Figures 2(a)–2(d)).

### 3.2. Construction and Evaluation of a Prognostic Model Based on the Risk Profiles of 3 AAMRGs

**Figure 3:**
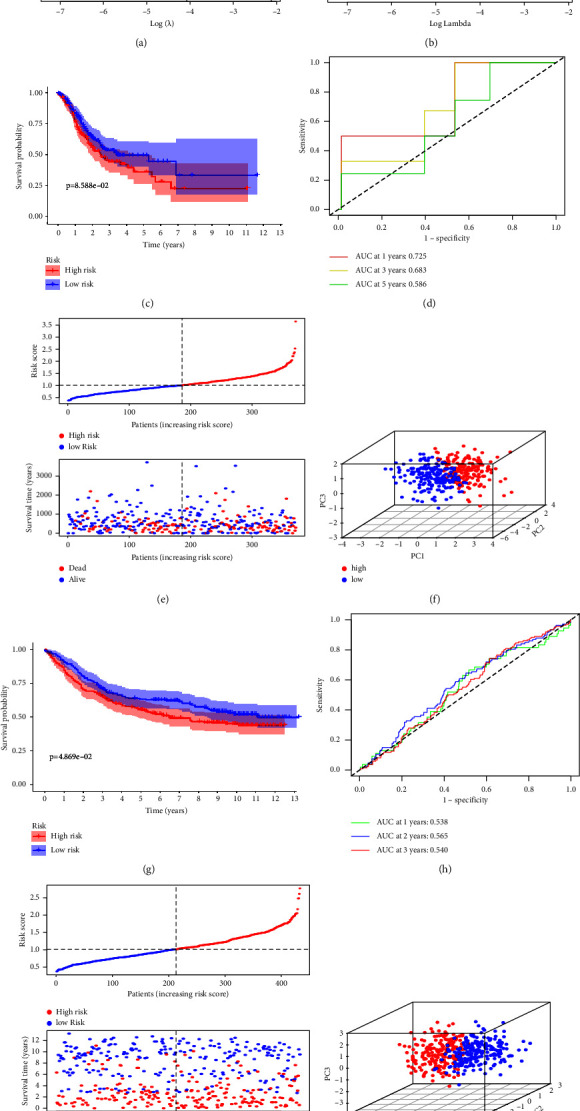
To construct a prognostic model of gastric cancer. (a) LASSO coefficient profiles are generated when a prognostic model is constructed by the LASSO algorithm. (b) Partial likelihood deviation plots are generated when a prognostic model is constructed by the LASSO algorithm and multivariate COX regression analysis. (c) The survival curves chart shows the difference in survival between the high-risk and low-risk patients in this prognostic model. (d) The accuracy of the prognosis model was tested by ROC curve. (e, f) The profiles of high-risk and low-risk groups in this prognostic model are shown. (g) Using the GSE84437 dataset to validate the prognostic model, the survival graph shows the difference in survival between the high-risk group and the low-risk group. (h) The ROC curve demonstrated the accuracy of this prognostic model in the GSE84437 dataset. (i-j) The GSE84437 dataset was used to show the profile of the high-risk and low-risk groups in the prognostic model.

The 16 AAMRGs were selected after differential expression analysis and univariate COX regression analysis based on a dataset of 407 STAD patients obtained from the TCGA database, which was combined with 480 AAMRGs from the GSEA database. From these genes, a 10-fold cross-validation by the “glmnet” software package and LASSO regression analysis were performed to screen for prognostically relevant AAMRGs. Finally, three core AAMRGs associated with prognosis were obtained by multivariate COX regression analysis and these were DNA methylation transferase 1 (DNMT1), prothrombin II (F2R), and GPX3. Based on these 3 genes, we constructed a prognostic amino acid metabolism risk model, generated LASSO coefficient profiles (Figure 3(a)) and partial likelihood deviation plots (Figure 3(b)) for the relevant core genes involved. We used the following formula to determine each STAD patient's risk score: risk score = (0.28 × F2R expression) + (−0.27 × DNMT1 expression) + (0.19 × GPX3 expression). Finally, based on the median risk score, the prognostic model was divided into two subgroups: high- and low-risk groups.

According to survival analysis, patients in the low-risk group outlived those in the high-risk group by a substantial margin (*P* < 0.01, Figure 3(c)). With respect to predicting the prognosis of STAD based on the three AAMRGs, the risk model's AUC values for the three genes were 0.725, 0.684, and 0.683 in the 1-, 2-, and 3-year ROC curves, respectively (Figure 3(d)). These values were highly sensitive and specific. The distribution of risk scores and the survival status of these three AAMRGs in the TCGA dataset are shown by the risk curves in Figure 3(e). The PCA analysis in Figure 3(f) then shows the distribution of risk scores for these 3 genes in the TCGA dataset. To validate the model's predictive performance, we used the GSE84437 dataset from the GEO database as an externally validated dataset and the 407 STAD patients from the test cohort as the validation cohort and calculated their risk scores using the above formula. As with the training cohort, the validation group was subgrouped based on the training group's risk score threshold, which showed that overall survival (OS) was worse in the low-risk group (*n* = 213) than that in the high-risk group (*n* = 220) (*P* < 0.01, Figure 3(g)). The AUC values at 1, 2, and 3 years were >0.5 (Figure 3(h)). The risk curves in Figure 3(i) demonstrate the distribution of risk scores and survival status of these 3 AAMRGs in the GSE84437 dataset. The PCA analysis in Figure 3(j) demonstrates the distribution of risk scores for these 3 genes in the GSE84437 dataset. All these results show that the prognostic model has good predictive ability and accuracy.

### 3.3. Evaluation and Validation of Biomarkers as Key Targets Influencing the Prognosis of Gastric Cancer

**Figure 4:**
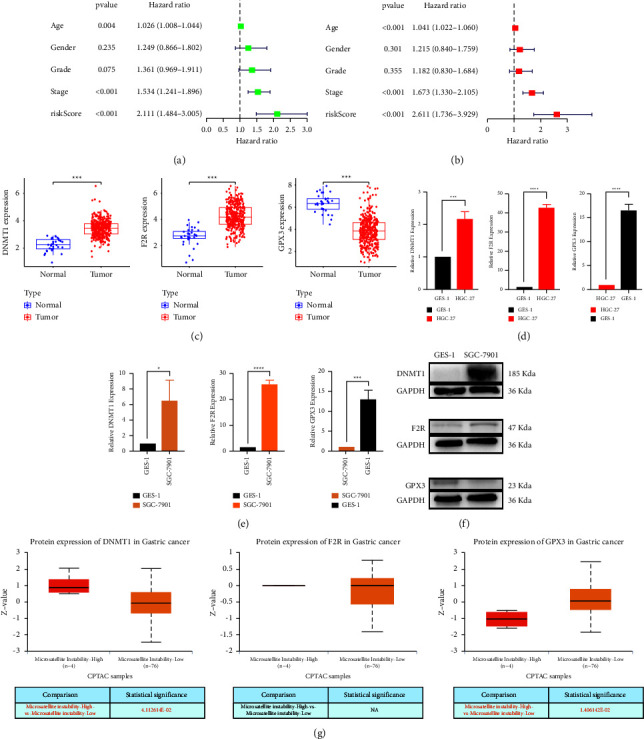
Evaluation and validation of key tumor markers. (a) Univariate COX regression analysis was used to evaluate the efect of this model on the prognosis of patients with gastric cancer. (b) Multivariate COX regression analysis was used to evaluate the effect of this model on the prognosis of gastric cancer patients. (c) LIMMA software packages were used to evaluate the diferential expression of the three AAMRGs in gastric cancer (^∗∗∗^*P* < 0.001). (d) qRT-PCR was used to detect the differential expression of the three AAMRGs in HGC-27 cells and GES-1 cells (^∗∗∗^*P* < 0.001, ^∗∗∗∗^*P* < 0.0001). (e) qRT-PCR was used to detect the diferential expression of the three AAMRGs in SGC-7901 cells and GES-1 cells (^∗^*P* < 0.05, ^∗∗∗^*P* < 0.001, ^∗∗∗∗^*P* < 0.0001). (f) Western blot was used to detect the differential expression of the three AAMRGs in SGC-7901 cells and GES-1 cells. (g) The differential expression of these three AAMRGs at the protein level was demonstrated by proteomic data from the CPTAC database.

Using this prognostic model, we performed univariate and multivariate Cox regression analyses (Figures 4(a) and 4(b)) to see whether the predictive values of the three genes from the screening (DNMT1, F2R, and GPX3) was unaffected by these clinical parameters. The results showed that the amino acid metabolism prognostic model constructed based on these three genes could be used as an independent prognostic factor for predicting the occurrence of STAD (HR > 1, *P* < 0.01). Subsequently, the box line plots were used to demonstrate the differential expression levels of these three AAMRGs in STAD and normal tissues (Figure 4(c)), and the results of qRT-PCR (Figures 4(d) and 4(e)) and western blotting (Figure 4(f)) validated the above analysis. We also used proteomics data from the CPTAC database to analyze the differential expression of these three AAMRGs at the protein level. The analysis results (Figure 4(g)) we obtained were generally consistent with the experimental results in Figure 4(f).

### 3.4. Exploration of Signaling Mechanisms Associated with Prognostic Models

**Figure 5:**
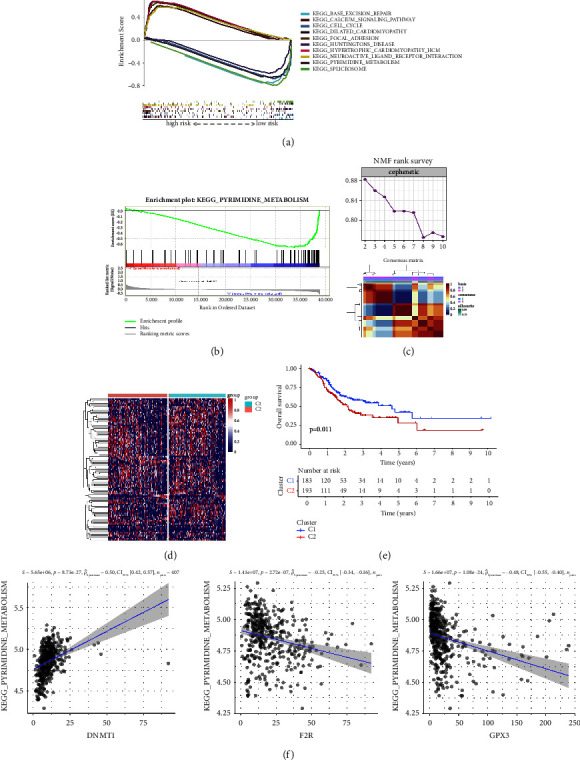
Pyrimidine metabolism is a key factor leading to poor prognosis in patients with gastric cancer. (a) Multi-GSEA enrichment analysis showed the results of enrichment analysis in the high-risk and low-risk groups in the prognostic model. (b) GSEA enrichment analysis showed that pyrimidine metabolism was related to the prognosis model of amino acid metabolism. (c) NMF ranking survey of unsupervised clustering results and consensus matrix heat map of two clusters was produced by unsupervised clustering. (d) Heatmap demonstrating the variability in expression between patients with Cluster 1 and Cluster 2. (e) Survival curve of Cluster 1 and Cluster 2 groups (*P*=0.011). (f) Correlation between expression of prognostic factors DNMT1, F2R, and GPX3 in STAD and pyrimidine metabolism.

We explored the potential molecular mechanisms by which risk scores influence tumor progression based on the biophenotypes of correlations involved in high- and low-risk groups in the prognostic models. The results of enrichment analysis by GSEA showed that prognostic model-related genes were significantly enriched in many pathways. The calcium-related signaling pathways, dilated cardiomyopathy, adhesive plaques, hypertrophic cardiomyopathy, and neuroactive ligand-receptor interactions may be associated with high-risk scores in the amino acid metabolism prognostic model (Figure 5(a)). In contrast, base excision repair, cell cycle, Huntington's disease, pyrimidine metabolism, and spliceosomes were associated with low-risk scores in the prognostic model.  Figure 5(b) demonstrates the correlation between the prognostic model of amino acid and pyrimidine metabolic processes. The NMF algorithm was then applied to a cluster of the TCGA-STAD cohort based on the enrichment scores of pyrimidine metabolism in GSEA, and based on the coefficients, we determined that the optimal *k* value was 2 (Figure 5(c)). The NMF clustering algorithm categorized patients with TCGA-STAD into cluster 1 and cluster 2. The heatmap demonstrates that the expression of patients with cluster 2 is overall slightly higher than that of patients with cluster 1 (Figure 5(d)). The clustering results showed that patients in cluster 2 had significantly shorter OS compared to those in cluster 1 (Figure 5(e)). This suggested that pyrimidine metabolism is a key factor contributing to the poor prognosis of STAD patients. Finally, we calculated the correlation between DNMT1, F2R, and GPX3, and pyrimidine metabolism based on ssGSEA scores derived from their profiles. It appears that DNMT1 has the strongest positive correlation with pyrimidine metabolism in STAD patients (*R* = 0.50, *P* < 0.01) and GPX3 has the strongest negative correlation (*R* = −0.48, *P* < 0.01; Figure 5(f)). Hu et al. [28] showed a link between the expression level of GPX3 and resistance to a chemotherapeutic drug, 5-fluorouracil (5-Fu), which can affect pyrimidine metabolism [29]. Therefore, we chose GPX3 as the target of interest for conducting further validation experiments.

### 3.5. Prognostic Columnar Plot of the AAMRG-Associated Risk Models

**Figure 6:**
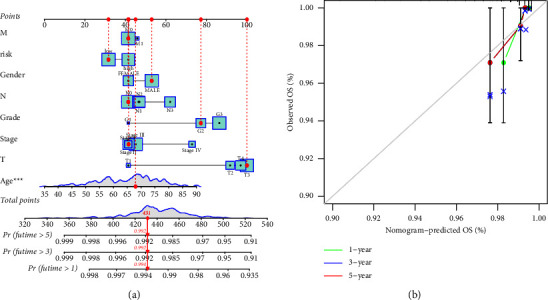
Prognostic nomogram combined with risk score model and clinicopathological features. (a) Age, stage, grade, TNM grade, and risk score histogram were used to predict 1-year, 3-year, and 5-year survival (^∗∗∗^*P* < 0.001). (b) 1-, 3-, and 5-year calibration curves of the TCGA data set.

We constructed a column-line plot of risk scores including age, stage, and TNM grading using the three AAMRGs to forecast the survival rates of STAD patients in the first, third, and fifth years (Figure 6(a)). The calibration curves in Figure 6(b) show that the predicted and current status of one-, three-, and five-year survival values are consistent.

### 3.6. GPX3 Affects STAD Cell Proliferation and Invasion

**Figure 7:**
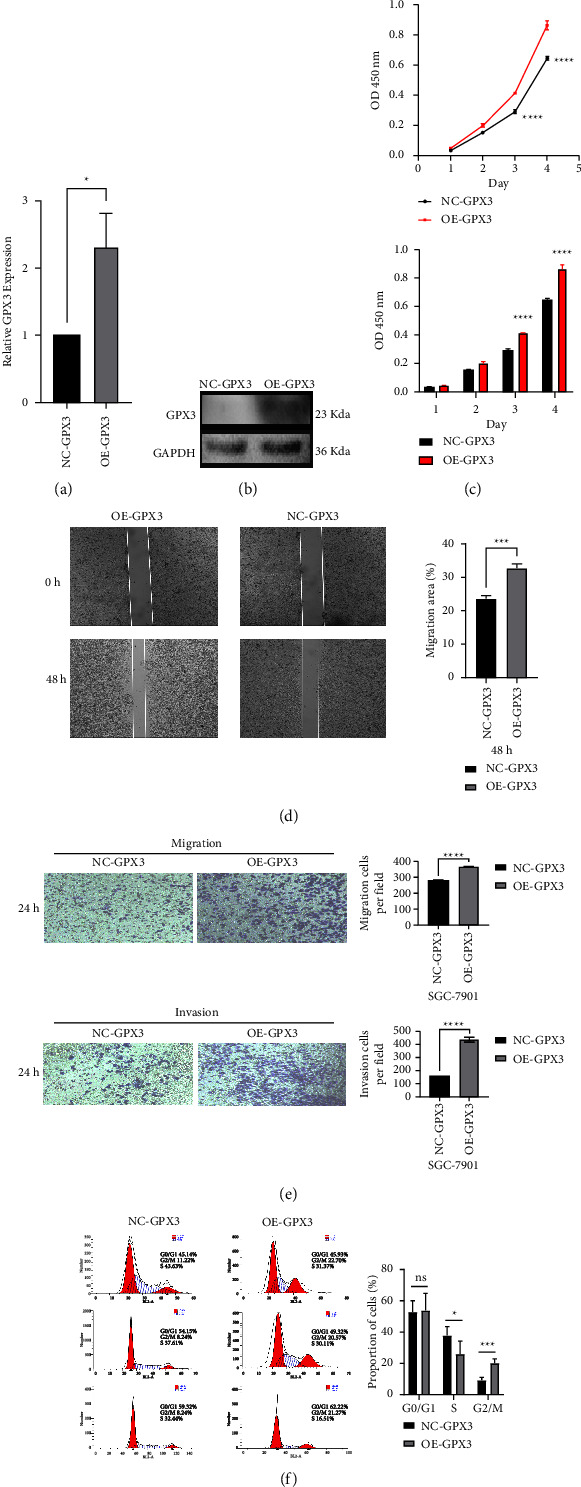
Effect of GPX3 on invasion and migration of STAD cells. (a, b) The expression level of GPX3 in STAD cells transfected with OE-GPX3 was analyzed by qRT-PCR and Western blot (^∗^*P* < 0.05). (c) The cell proliferation ability of the untransfected group (NC-GPX3) and the transfected overexpression plasmid group (OE-GPX3) was detected by the CCK8 assay in SGC-7901 cells (^∗∗∗∗^*P* < 0.0001). (d) Trough wound healing assays, we examined the efect of GPX3 expression level on cell migration ability (^∗∗∗^*P* < 0.001). (e) Representative data from Transwell migration and Matrigel invasion assays performed with the GPX3 is overexpressed (^∗∗∗∗^*P* < 0.0001). (f) Te changes of cell cycle between NC-GPX3 and OE-GPX3 group were detected by fow cytometry. When GPX3 is overexpressed, the proportion of G2/M phase cells increases (^∗^*P* < 0.05, ^∗∗∗^*P* < 0.001).

In the aforementioned investigation, we used the western blot assay and qRT-PCR to determine the GPX3 expression levels in cell lines (HGC-27, SGC-7901, and GES-1) associated with STAD. It was found that, in comparison to the normal gastric mucosal epithelial cell line, GES-1, GPX3 expression levels were reduced in HGC-27 and SGC-7901 cells (Figures 4(d) and 4(e)). To further investigate the biological role of GPX3 in STAD cells, we upregulated the expression level of GPX3 in SGC-7901 cells by transfecting them with the overexpression plasmid of GPX3 (OE-GPX3). We measured the overexpression efficiency of this plasmid by qRT-PCR and western blotting (Figures 7(a) and 7(b)). We confirmed by using CCK8 assays that the expression level of GPX3 could significantly affect the proliferative capacity of STAD cells and that the SGC-7901 cells were more proliferative after transfection with the overexpression plasmid (Figures 7(c) and 7(d)). Then, the impact of GPX3 on the migration and invasion capacity of STAD cells were examined using the transwell and wound healing assays. These demonstrated that overexpression of GPX3 markedly improved the migration and invasion capacity of SGC-7901 cells (Figure 7(e)). Flow cytometry results further indicated that the GPX3 overexpression group of cells (OE-GPX3) had an increased proportion of cells at the G2/M stage when compared to the control STAD cell group (NC-GPX3) (Figure 7(f)). Overall, these results suggested that GPX3 can serve as an independent biological prognostic marker for STAD, where it appears to influence the proliferation and invasion of these cancer cells.

### 3.7. GPX3 Inhibits Pyrimidine Metabolism Levels in STAD

**Figure 8:**
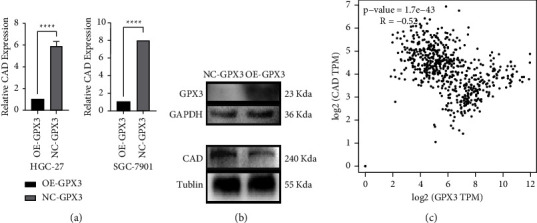
Effect of GPX3 on pyrimidine metabolism. (a) qRT-PCR was used to detect the efect of GPX3 expression on CAD in HGC-27 and SGC-7901 cells (^∗∗∗∗^*P* < 0.0001). (b) Western blot was used to detect the efect of GPX3 expression on CAD in SGC-7901 cells. (c) Te correlation between GPX3 and CAD expression level was analyzed through TIMER2.0 online database.

We calculated the correlation between GPX3 and pyrimidine metabolism by calculating the ssGSEA score. The results indicated that GPX3 plays an inhibitory role with respect to the level of pyrimidine metabolism in STAD (Figure 5(e)). Thus, changes in the expression of pyrimidine metabolism-related markers could be detected when the level of GPX3 was altered in STAD cells. Carbamoylphosphate synthase 2 (CAD), aspartate transcarbamoylase (ATC), and dihydrolactamase (DHA) form a multifunctional protein complex within cells that participates in the initial three rate-limiting steps of pyrimidine nucleotide synthesis, and it also plays a key role in the initial synthesis of pyrimidine nucleotides [30]. We detected a significant downregulation of CAD expression levels with the upregulation of GPX3 expression in GPX3 overexpression of HGC-27 and SGC-7901 cells as measured by qRT-PCR and western blotting (Figures 8(a) and 8(b)). In addition, we analyzed the correlation between GPX3 and CAD in STAD using the TIMER 2.0 online database and found that GPX3 was negatively correlated with CAD (*R* = −0.52) (Figure 8(c)). This was consistent with our experimental results, thereby further demonstrating that GPX3 exerts an inhibitory effect on the level of pyrimidine metabolism in STAD.

### 3.8. Exploration of the Mechanism by Which GPX3 Affects 5-Fu Resistance

**Figure 9:**
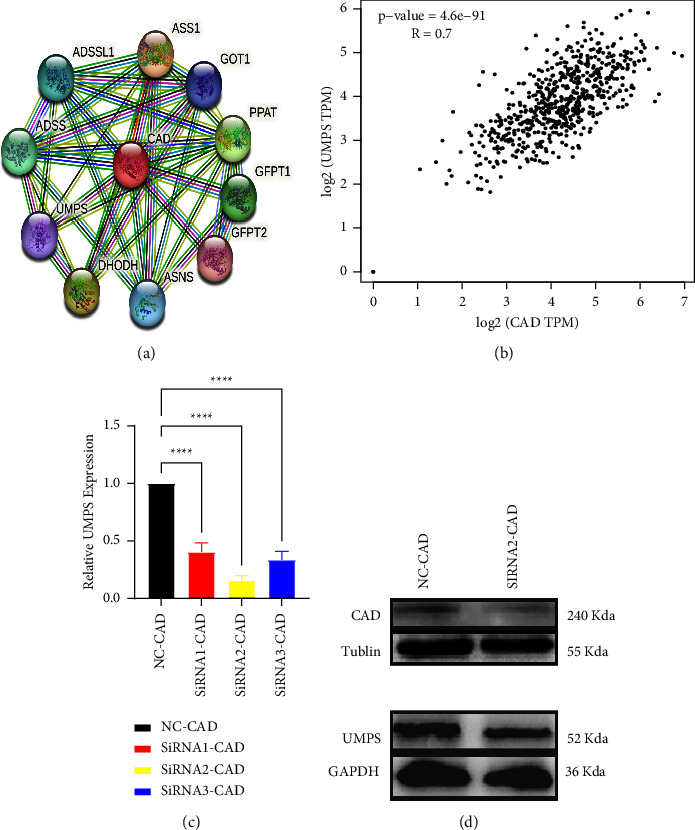
Effect of CAD on 5-Fu. (a) PPI network built directly related to CAD through a STRING database. (b) The correlation between UMPS and CAD expression level was analyzed through TIMER2.0 online database. (c) The knockdown effciency of CAD in SGC-7901 cells was detected by qRT-PCR (^∗∗∗∗^*P* < 0.0001). (d) The efect of CAD expression on UMPS expression in SGC-7901 cells was detected by western blot assay.

Hu et al. [28] found an association between GPX3 and drug sensitivity to the antitumor drug, 5-Fu. Moreover, our experimental results demonstrated that GPX3 may affect 5-Fu resistance in STAD by affecting the expression of CAD and inhibiting the level of pyrimidine metabolism. However, the mechanism whereby how CAD affects 5-Fu resistance is ongoing. Uridine-phosphate synthase (UMPS) is a bifunctional enzyme in humans that functionally catalyzes the last two steps of the pyrimidine biosynthetic process [31]. It has been shown that when 5-Fu exerts its antitumor mechanism of action, UMPS can convert a part of 5-Fu into components with cytotoxicity, thus inhibiting the activity of the proapoptotic protein, Bok. This allows cancer cells in a nonproliferative state to escape the damaging properties of 5-Fu and develop secondary drug resistance [32–35]. Yu et al. also verified the modulation of 5-Fu drug sensitivity by UMPS expression through their findings [36]. Therefore, we first constructed a PPI network associated with CAD through the STRING database, and this showed a correlation between it and UMPS (Figure 9(a)). Subsequently, we verified the correlation between CAD and UMPS in STAD using the TIMER 2.0 online database (*R* = 0.7) (Figure 9(b)). We then found that in SGC-7901 cells, both CAD and UMPS expression were downregulated, by using qRT-PCR and western blotting analysis (Figures 9(c) and 9(d)). Therefore, based our results and others in the literature, we suggest that GPX3 plays a significant role in pyrimidine metabolism, possibly through the CAD-UMPS signaling axis, in influencing 5-Fu resistance.

### 3.9. GPX3 Mediates the AMPK/mTOR Pathway in STAD

**Figure 10:**
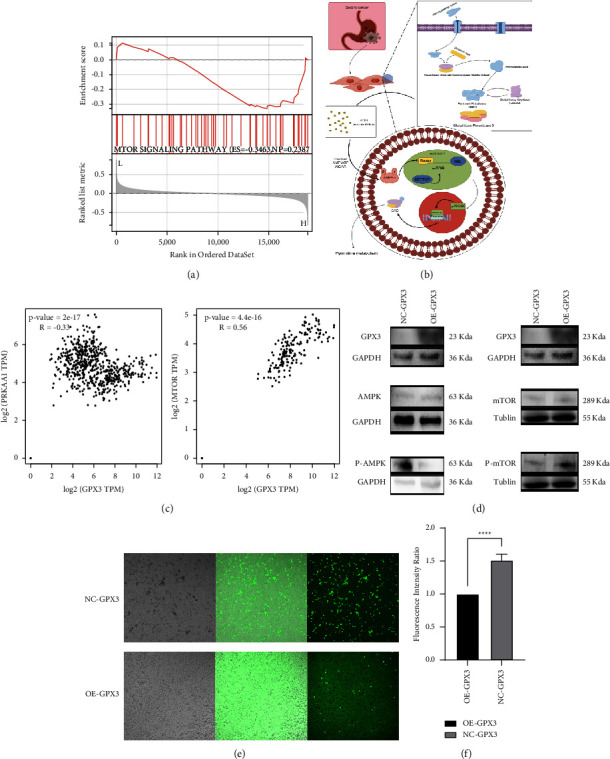
Effect of GPX3 on the AMPK/mTOR signaling pathway. (a) Using Sangerbox 3.0 tool, we performed GSEA enrichment analysis for GPX3. The results show that GPX3 is related to mTOR signaling pathway. (b) Specifc mechanism of GPX3 regulation of pyrimidine metabolism through AMPK/mTOR signaling pathway (picture design by Figdraw). (c) The correlation between GPX3 and the expression level of AMPKa1 and mTOR was analyzed through TIMER2.0 online database. (d) The effect of GPX3 expression on AMPKa1 and mTOR expression in SGC-7901 cells was detected by western blot analysis. (e) Laser confocal microscopy was used to show the diference of ROS content between the NC-GPX3 group and the OE-GPX3 group. (f) The ROS changes in the NC-GPX3 and OE-GPX3 group were quantitatively detected by fuorescent enzyme labeling (^∗∗∗∗^*P* < 0.0001).

GPX3 is known to play a key role in regulating the levels of ROS in vivo [37]. The level of ROS in the body is influenced by the expression activity of AMPK*α* via its signaling pathway [38]. We performed GSEA enrichment analysis of single genes by using the Sangerbox 3.0 tool, targeting GPX3. The GSEA data indicated a connection between GPX3 and the mTOR signaling pathway (Figure 10(a)). Ben-Sahra et al. also found that the downstream mTORC1 receptor in the mTOR signaling pathway [39] can alter the S6K kinase activity downstream of the pathway, affecting the expression level of CAD and the process of pyrimidine synthesis. Therefore, we reasonably hypothesized that GPX3, in STAD, may influence the level of pyrimidine metabolism by regulating the amount of ROS in the organism and this is mediated by the AMPK/mTOR signaling pathway (Figure 10(b)).

To confirm the regulatory role of GPX3 in the AMPK/mTOR signaling pathway, we used western blotting and ROS detection kits to verify this hypothesis and selected AMPKa1 and mTOR as markers of the AMPK/mTOR pathway. We first used the TIMER 2.0 online database to analyze the correlation of GPX3 with AMPKa1 and mTOR, and this showed that GPX3 showed a positive correlation (*R* = 0.56) with mTOR but a negative correlation (*R* = −0.33) with AMPKa1 (Figure 10(c)). By western blotting analysis, we determined that after overexpression of GPX3 in SGC-7901 cells, the protein level of phospho-AMPK was greatly suppressed, and the protein level of phospho-mTOR was markedly raised. In contrast, the total amount of AMPK and mTOR expression did not change (Figure 10(d)). We also observed that the ROS content in GPX3 overexpressing SGC-7901 cells (OE-GPX3) was significantly lower than that in normal SGC-7901 cells (NC-GPX3) by laser confocal microscopy and a fluorescent enzyme marker (Figures 10(e) and 10(f)).

Based on these results, we hypothesized that GPX3 mediates the AMPK/mTOR signaling pathway by regulating the level of ROS in tumor cells and triggering an imbalance of oxidative stress in the body, which subsequently has an impact on the level of pyrimidine metabolism and 5-Fu resistance in patients with STAD.

## 4. Discussion

Tumor cells have a higher requirement for exogenous amino acids when compared to normal cells. Hence, alterations in amino acid metabolism can have a significant impact on tumor cells and the tumor immune microenvironment [40–44]. Clinical induction of cancer cell apoptosis by amino acid depletion therapy has also become a research hotspot in recent years [45]. Therefore, we use AAMRGs as the target of STAD treatment in order to explore their influence on the mechanism of this disease with a view to providing potential new avenues for tumor research.

The study of cancer biology has advanced significantly in recent decades due to the introduction of high-throughput sequencing [46]. Among them, bioinformatics, as one of the main tools for detecting potential prognostic biomarkers in cancer-related fields, has helped to screen more biomolecules involved in tumor progression [47]. Thus, our goal was to identify a predictor of amino acid metabolism affecting STAD metastasis and poor prognosis by using the bioinformatics approach. In addition, we assessed the effect of amino acid metabolism on the relevant biological functions in tumors by exploring their specific molecular mechanism in STAD cells. We first screened 16 AAMRGs which affected the prognosis of STAD patients by analyzing the TCGA-STAD cohort dataset using differential expression and Kaplan–Meier analyses. LASSO and multivariate COX regression analyses were then used, and three independent prognostic variables (DNMT1, F2R, and GPX3) were found. A prognosis risk model for STAD was then built using these findings. Based on the prognostic model's median risk score, the TCGA-STAD samples were divided into low- and high-risk groups. GSEA enrichment analysis revealed that pyrimidine metabolism was one of the most enriched gene features in the low-risk population and that the poor prognosis of STAD patients was primarily associated with these heterocyclic compounds.

Clinical pyrimidine-related antimetabolites have previously been used to treat tumor-like diseases [48], and these drugs regulate the level of pyrimidine metabolism in the body through the action of certain key enzymes [49–51]. He et al. demonstrated that the expression of CAD, a key enzyme in de novo synthesis of pyrimidine, plays a critical role in chemotherapy resistance in STAD [52]. When the pyrimidine biosynthetic pathway was blocked, the sensitivity of the drug, 5-Fu, to cancer cells, changed [53, 54]. We used NMF clustering analysis to identify the genes relevant to pyrimidine metabolism. Then, we used this information to separate the TCGA-STAD samples into two groups depending on how the genes were associated to pyrimidine metabolism. The OS rates of the two clusters were compared using Kaplan–Meier analysis. Others have shown that high pyrimidine metabolism levels are linked to a worse prognosis for STAD patients.

To clarify the connection between DNMT1, F2R, and GPX3 expression and pyrimidine metabolism, we performed ssGSEA analysis using pyrimidine metabolism-derived features. The ssGSEA score derived from pyrimidine metabolism characterization showed the strongest negative correlation between GPX3 and pyrimidine metabolism in STAD. We then constructed a GPX3-overexpressed SGC-7901 cell line by transfection of GPX3 into an overexpression plasmid and employed western blotting and qRT-PCR measurements to confirm the transfection efficiency. We found that GPX3 overexpression markedly increased the migration and invasion of STAD cells by using the scratch test and transwell assays. Concurrently, there was an upregulation of GPX3 expression and a downregulation of CAD, P-AMPKa1, and P-mTOR expression. Within pyrimidine metabolism, qRT-PCR and western blotting measurements demonstrated a positive correlation between CAD and UMPS expression. In addition, the effect of GPX3 on 5-Fu resistance may also be linked to the CAD-UMPS signaling axis. These findings strongly suggest that GPX3 is an important regulator in STAD, affecting the level of pyrimidine metabolism and 5-Fu resistance, and it may also be related to the AMPK/mTOR signaling pathway. However, the dual role of ROS in cancer, especially their paradoxical ability to induce proliferation or apoptosis in cancer cells, has led to disappointing results in the application of antioxidants in clinical cancer therapy. Thus, in relevant experimental studies, GPX3 sometimes exerts opposite effects on tumor cells due to different intracellular ROS concentrations. How to regulate the intracellular ROS concentration in the organism so as to exert the desired effect of antioxidants in inhibiting the proliferation of tumor cells is a question that we need to keep exploring in subsequent studies.

In conclusion, we found that GPX3 could not only function as a key target of amino acid metabolism in STAD but also GPX3 could be used as a prognostic biomarker in patients with STAD. GPX3 inhibited the level of pyrimidine metabolism in STAD cells via the ROS/AMPK/mTOR signaling pathway, which could affect the migration and invasive ability of these cells. These results could eventually lead to improved approaches for treating and diagnosing stomach cancer and provide ways to lower the antidrug resistance experienced in some patients.

## Data Availability

The data used to support the findings of this study are included within the article.
